# miR-514a promotes neuronal development in human iPSC-derived neurons

**DOI:** 10.3389/fcell.2023.1096463

**Published:** 2023-02-07

**Authors:** Yuichi Akaba, Satoru Takahashi, Keiichiro Suzuki, Kenjiro Kosaki, Keita Tsujimura

**Affiliations:** ^1^ Group of Brain Function and Development, Neuroscience Institute of the Graduate School of Science, Nagoya University, Nagoya, Aichi, Japan; ^2^ Research Unit for Developmental Disorders, Institute for Advanced Research, Nagoya University, Nagoya, Aichi, Japan; ^3^ Department of Pediatrics, Asahikawa Medical University, Asahikawa, Hokkaido, Japan; ^4^ Institute for Advanced Co-Creation Studies, Osaka University, Osaka, Japan; ^5^ Graduate School of Engineering Science, Osaka University, Osaka, Japan; ^6^ Graduate School of Frontier Bioscience, Osaka University, Osaka, Japan; ^7^ Center for Medical Genetics, Keio University School of Medicine, Tokyo, Japan

**Keywords:** microRNA, miR-514a, IPSC, neuronal development, dendrite, mTOR

## Abstract

Proper development and function of the central nervous system require precise regulation of gene expression. MicroRNAs (miRNAs), a group of small non-coding RNAs that can negatively regulate gene expression at the post-transcriptional level, are critical regulators of neuronal development, and dysregulation of microRNAs has been implicated in various neurological disorders. Changes in microRNA expression and repertoire are related to the emergence of social and behavioral variations in closely related primates, including humans, during evolution. MicroRNA-514a (miR-514a) is an X-linked miRNA that is conserved in species with higher social and cognitive functions, and frequent tandem duplications of miR-514a have been found in primate genomes. Here, we demonstrate that miR-514a plays a crucial role in neuronal development in neurons derived from human induced pluripotent stem cells (iPSCs). Overexpression of miR-514a increased dendritic length, soma size, and activity levels of mammalian target of rapamycin (mTOR) signaling in induced pluripotent stem cell-derived neurons, whereas blocking of endogenous miR-514a inhibited neuronal development. Furthermore, we performed a functional analysis of the miR-514a variation found during primate evolution, to investigate the impact of miR-514a sequence variation and associated changes in expression on brain development during evolution. We found that mutation in miR-514a significantly reduced the expression of the mature form and abolished the effects observed when native miR-514a was expressed. Our findings provide new insights into the functional role of miR-514a in the regulation of neuronal development and evolution of primate brain development.

## 1 Introduction

The functional development of the brain depends on the formation of precise neural circuitry constructed by the proper development of neurons, such as neuritogenesis and neuronal cell growth. Multiple lines of evidence have shown that adequate regulation of gene expression is fundamental to neuronal development and that its dysregulation causes various neurological diseases (Riccio, 2010; Penzes et al., 2011; de la Torre-Ubieta et al., 2016; Willsey et al., 2018; Campbell and Wood, 2019).

MicroRNAs (miRNAs) are small single-stranded non-coding RNAs composed of 18–22 nucleotides that can negatively regulate gene expression at the post-transcriptional level and thereby influence many biological processes. miRNA genes are primarily transcribed by RNA polymerase II to become primary miRNAs (pri-miRNAs). Pri-miRNAs are further processed by the Drosha/DGCR8 complex in the nucleus to form precursor miRNAs (pre-miRNAs). Pre-miRNAs are exported to the cytoplasm and cleaved by Dicer to produce mature miRNAs. The mature guide strand is then bound to Argonaute proteins to form the RNA-induced silencing complex (RISC) and binds to the 3′untranslated region (3′UTR) of target messenger RNAs (mRNAs), leading to translational repression or mRNA degradation (Winter et al., 2009; Gebert and MacRae, 2019). Recent studies have shown that miRNAs play an important role in the regulation of neuronal development (Xu et al., 2010; Olde Loohuis et al., 2012). MicroRNA-379/410 (miR-379/410) promotes neural stem cell differentiation and neuronal migration in the neocortex by repressing N-cadherin expression (Rago et al., 2014). miR-17/92 has also been implicated in axon outgrowth and guidance *via* downregulation of PTEN, leading to the activation of the mammalian target of rapamycin (mTOR) pathway (Zhang et al., 2013). miR-379/410 controls dendritic cell morphology by regulating Pumilio2 (Fiore et al., 2009). Additionally, accumulating evidence suggests that miRNA dysfunction contributes to various neurological disorders (Xu et al., 2010). A recent study reported that miR-34 is downregulated in Parkinson’s disease, reducing the levels of DJ-1 and Parkin in the brain, two proteins involved in the ubiquitin proteasome system in neurons, causing cell death (Rezaei et al., 2021). Moreover, mutations in miRNA genes are associated with development and progression of several human diseases. The gain-of-function mutation in miR-140 leads to the repression of mutant miR-140 targets through abundant expression of mutant miR-140 without defects in miRNA processing, leading to human skeletal dysplasia (Grigelioniene et al., 2019). The miR-218 mutation identified by screening amyotrophic lateral sclerosis (ALS) genomes causes inhibition of miR-218 biogenesis due to reduced dicer processing, resulting in dysregulation of motor neuron activity (Reichenstein et al., 2019).

miRNAs can be classified into two groups, conserved and non-conserved miRNAs, based on their species sequence conservation (Bentwich et al., 2005). Most studies have focused on highly conserved miRNAs (Kontarakis et al., 2018; Lyu et al., 2021). However, non-conserved miRNAs represent a potentially important source of functional novelty during evolution. Non-conserved miRNAs can be indicators of adaptation in the genome of an organism, leading to novel phenotypes and a number of diseases (McCreight et al., 2017). miR-941, a human-specific miRNA, could be associated with hedgehog and insulin signaling pathways, thus potentially playing a role in the evolution of human longevity and cognition (Hu et al., 2012). miR-95, whose conservation is limited to the primate lineage and a few higher mammals, represses SUMF1 to disrupt lysosomal function, and is associated with lysosomal storage disorder (Frankel et al., 2014).

Changes in miRNA expression and repertoire are related to the emergence of social and behavioral variations in closely related primates, including humans, during evolution. miR-514a is a member of the non-conserved X-linked miRNA cluster in primates and has different copy numbers among species (Zhang et al., 2007). For example, miR-514a has three copies in humans, four copies in chimpanzees, one copy in other primate species, but no copies in rodents. Phylogenetic analysis of miR-514a indicated that duplications occur independently in humans and chimpanzees (Zhang et al., 2007). Several studies have indicated that miR-514a contributes to oncogenic alterations in various cancers. miR-514a represses proliferation in ovarian cancer cells by targeting the ATP-binding cassette subfamily (Xiao et al., 2018), whereas the aberrant expression of miR-514a is upregulated in gastric cancer tissues (Liu et al., 2014).

In this study, we identified a novel function of miR-514a in central nervous system (CNS) development, which has not yet been clarified. Here, we show that miR-514a plays a key role in neuronal development in neurons derived from human induced pluripotent stem cells (iPSCs). Overexpression of miR-514a promoted dendritic development and upregulated the mTOR signaling pathway, whereas loss-of-function of miR-514a had the opposite effects. Furthermore, we performed a functional analysis of the miR-514a variation found during primate evolution to investigate the impact of miR-514a sequence variation and associated changes in expression on brain development during evolution. We found that mutation in miR-514a significantly reduced the expression of the mature form and abolished the effects observed when native miR-514a was expressed. These results suggest that miR-514a is an important factor in neuronal development and evolution of primate brain development.

## 2 Materials and methods

### 2.1 Cell culture

Human iPSC lines 201B7 (HPS0063), 585A1 (HPS0354), and 648A1 (HPS0360) were provided by the Riken BioResource Research Center (Tsukuba, Japan) (Takahashi et al., 2007; Okita et al., 2013). iPSCs were cultured on a feeder cell layer of mitomycin C-treated MEF (Kumazaki et al., 2011). The feeder dishes were prepared by seeding thawed feeder cells into gelatin-coated dishes. iPSCs were cultured in the medium for human embryonic stem cells (ESCs) [DMEM/F12 (Gibco) supplemented with 15% knockout serum replacement (Gibco, Billings, MT, United States), 2 mM glutamine (Gibco), 1 mM 2-mercaptoethanol (Gibco), 1× non-essential amino acids (Gibco), 4 ng/mL bFGF (Wako, Osaka, Japan), 100 units/mL penicillin and 100 μg/mL streptomycin]. Cells were incubated in an incubator with 5% CO_2_ and 95% air at 37°C in a humidified atmosphere.

### 2.2 Neuronal differentiation

Neuronal differentiation of human iPSCs was induced as previously described (Arioka et al., 2018). Briefly, iPSCs were pretreated with 3 µM SB431542 (Cayman, Ann Arbor, MI, United States), 3 µM CHIR99021 (Cayman), and 3 µM dorsomorphin (Sigma, Burlington, MA, United States) for 1 week (day 0–7) and dissociated into single cells by incubation with TripLE Select (Gibco) for 5 min. The cells were cultured in neurosphere medium containing MHM [DMEM/F12, supplemented with 1× N2 (Gibco), 0.6% glucose (Sigma), 5 mM HEPES (Sigma), 100 units/mL penicillin and 100 μg/mL streptomycin, 1× B27 (Gibco), 20 ng/mL bFGF, 10 ng/mL hLIF (Nacalai, Kyoto, Japan), 10 µM Y-27632 (Cayman), 3 µM CHIR99021, 2 μM SB431542, 100 ng/mL FGF8b (Peprotech, Cranbury, NJ, United States), and 1 µM purmorphamine (Cayman) for 1 week (day 7–14)]. On day 14, neurospheres were passaged by dissociation into single cells in the same manner as primary neurosphere formation. For terminal differentiation into dopaminergic neurons, secondary neurospheres were dissociated 1 week after passage (day 21) and plated onto poly-L-ornithine/laminin/fibronectin-coated dishes at the density of 3 × 10^4^ cells/cm^2^ in dopaminergic neuron medium [MHM supplemented with B27, 10 µM DAPT (Cayman), 20 ng/mL BDNF (Peprotech), 20 ng/mL GDNF (R&D), 0.2 mM ascorbic acid (Sigma), 1 ng/mL TGF-β3 (R&D), and 0.5 mM dbcAMP (Sigma)].

### 2.3 Constructs

Lentiviral vectors expressing scrambled shRNA [pLV-control], miR-514a [pLV-hsa-precursor-miR-514a-3], miR-514a mutant-1 [pLV-U6>hsa-precursor-miR-514a-3G>A], miR-514a mutant-2 [pLV-U6>hsa-precursor-miR-514a-3 U(T)>C], miR-514a mutant-3 [pLV-U6>hsa-precursor-miR-514a-3 A>G], miR-514a mutant-4 [pLV-U6>hsa-precursor-miR-514a-3 U(T)>C], miR-514a mutant-5 [pLV-U6>hsa-precursor-miR-514a-3C > U(T)] and sponges against miR-514a-5p or -3p [pLV-U6>sponge-miR-514a-5pi or -3pi] were purchased from VectorBuilder (Chicago, IL, United States). The sequence of sponges were sponge-miR-514a-5pi: 5′-CAT​GAT​TGT​ACA​GCT​CCA​GAG​TAC​ATG​ATT​GTA​CAG​CTC​CAG​AGT​ACA​TGA​TTG​TAC​AGC​TCC​AGA​GTA-3′ and sponge-miR-514a-3pi: 5′-TCT​ACT​CAA​CAG​AGT​GTC​AAT​TCT​ACT​CAA​CAG​AGT​GTC​AAT​TCT​ACT​CAA​CAG​AGT​GTC​AAT-3′.

### 2.4 Lentivirus production

Lentiviruses were produced as previously described (Tsujimura et al., 2015). Briefly, lentiviral particles were constructed by co-transfection of HEK293T cells with lentivirus vector constructs pCMV-VSV-G-RSV-Rev and pCAG-HIVgp using polyethyleneimine (Polysciences, Warrington, PA, United States). The culture supernatants were collected 48 h after transfection, and viruses were introduced into the neurons by adding these supernatants to the culture media.

### 2.5 Immunocytochemistry

Cells were fixed on indicated days *in vitro* (DIV) with 4% paraformaldehyde in phosphate-buffered saline (PBS). The cells were washed with PBS, permeabilized, and blocked with blocking buffer (3% FBS and 0.1% Triton X-100 (Sigma) in PBS) at room temperature (23°C–26°C) for 30 min. The cells were incubated with the primary antibody solution overnight at 4°C. After washing with PBS, the cells were incubated with a secondary antibody solution at room temperature for 3 h, and mounted on glass slides after further washing with PBS. For quantification, multiple random fields were imaged from at least three independent differentiations.

### 2.6 Western blot

Total protein extracts were prepared by homogenizing cultured cells using lysis buffer containing 0.5% Nonidet P-40, 10 mM Tris-HCl, pH 7.5, 150 mM NaCl, and 1% protease inhibitor mixture (Nacalai). Whole-cell extracts were subjected to SDS-PAGE for electrophoresis and transferred to PVDF membranes (Merck Millipore, Darmstadt, Germany). The blots were blocked with Blocking-One (Nacalai) and incubated with the primary antibody solution overnight at 4°C. The membranes were incubated with the corresponding horseradish peroxidase-conjugated secondary antibody solution for 3 h at room temperature. Immunoreactive bands were detected by enhanced chemiluminescence using ECL Prime Western blotting detection reagent (GE Healthcare, Milwaukee, WI, United States). The intensities of the bands were quantified using ImageJ software.

### 2.7 Antibodies

The following primary antibodies were used for immunocytochemistry: monoclonal mouse antibody β-Ⅲ tubulin (Tuj-1) (1:1,000; Sigma), polyclonal guinea pig antibody microtubule-associated protein 2 (MAP2) (1:1,000; Synaptic Systems, Goettingen, Germany) and polyclonal rabbit antibody phospho-S6 ribosomal protein (pS6) Ser 235/236 (1:1,000; Cell Signaling Technology, Danvers, MA, United States). The following secondary antibodies were used: CF647 donkey anti-guinea pig IgG (H + L) highly cross-adsorbed (1:1,000; Biotium) and CF555 donkey anti-rabbit IgG (H + L) highly cross-adsorbed (1:1,000; Invitrogen). Nuclei were stained with Hoechst 33,258 (1:1,000; Wako).

The following primary antibodies were used for western blotting: polyclonal rabbit antibody pS6 Ser 235/236 (1:500; Cell Signaling Technology) and monoclonal rabbit antibody β-actin (1:500; Cell Signaling Technology). The following secondary antibodies were used: anti-rabbit IgG horseradish peroxidase linked whole antibody from donkey (1:10,000; GE Healthcare).

### 2.8 Morphological analysis of cultured neurons

Cultured neurospheres were infected with lentivirus at the stage of dissociation and plating for terminal differentiation into neurons (day 21 of differentiation). Seven days after transduction, differentiated neurons were fixed and immunostained for morphological analysis.

Neuronal differentiation was analyzed by counting cells on images of Tuj-1, MAP2 and Hoechst 33,258 stained cultures at 7 DIV. The percentages of Tuj-1 positive in Hoechst positive cells, and MAP2 positive in Hoechst positive cells were calculated to evaluate the rates of newly generated neurons.

To analyze dendritic development *in vitro*, neurons were subjected to immunostaining with an antibody against MAP2 at 7 DIV. Dendrites are defined as MAP2 positive neurites. Quantification of dendrite length was performed using ImageJ software.

The neuronal soma size *in vitro* was analyzed as previously described (Tsujimura et al., 2015). The neurons were immunostained with an antibody against MAP2 at 7 DIV. Images were examined using ImageJ software after manual delineation of the soma margins.

To analyze mTOR activity *in vitro*, after immunostaining with an antibody against pS6 at 7 DIV, cell counts were performed on the acquired images. We counted pS6 positive cells and the number of cells and calculated the percentage of pS6 positive cells.

### 2.9 Quantitative reverse-transcription polymerase chain reaction (qRT-PCR)

Total RNA was extracted using the mirVana miRNA Isolation Kit (Ambion) and subjected to reverse transcription using the TaqMan MicroRNA Reverse Transcription Kit (Applied Biosystems), according to the manufacturer’s protocol. qRT-PCR was performed using a StepOne real-time PCR system (Applied Biosystems). To detect mature miRNAs, a TaqMan microRNA assay kit (Applied Biosystems) was used, following the manufacturer’s protocol. Data were analyzed using the comparative Ct method. Values were normalized to those of U6 snRNA.

### 2.10 Statistical analysis

The results are presented as the average of at least three experiments, each performed in triplicate with standard errors. Statistical analyses were performed using Student’s *t*-tests (unpaired, two-tailed), and one-way ANOVA, followed by Tukey’s multiple comparison tests, as appropriate, using Prism 7 (GraphPad Software, San Diego, CA, United States). All data are presented as the mean ± standard error of the mean. Statistical significance was set at a *p*-value of <0.05.

## 3 Results

### 3.1 Structure and expression of miR-514a

**FIGURE 1:**
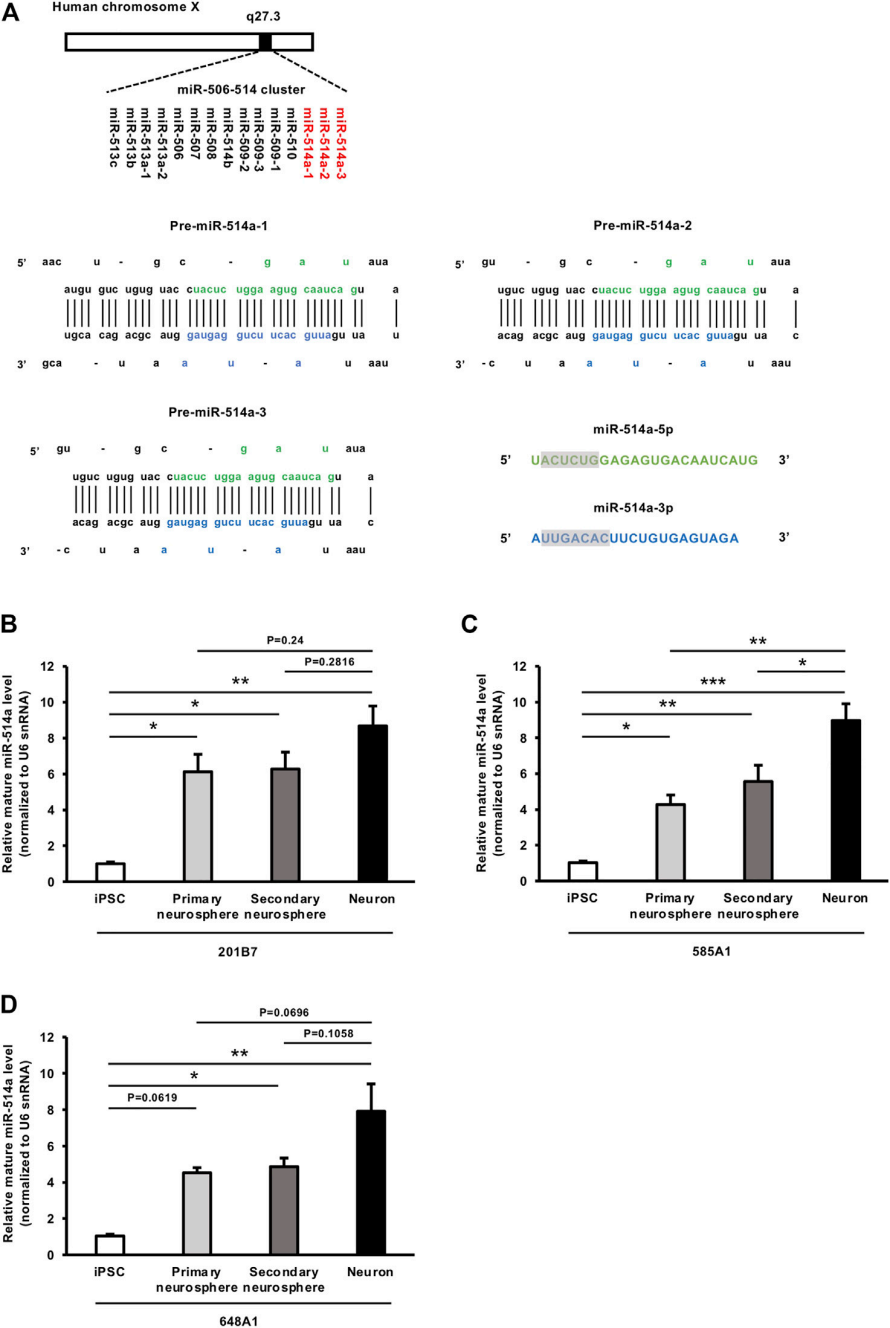
Genomic structure of miR-514a and its expression levels at each stage of neuronal differentiation. **(A)** Diagram of the structure of the human miR-514a locus and sequence of the precursor of miR-514a (pre-miR-514a). miR-514a belongs to the X-linked primate-specific miR-506-514 cluster. Primary miR-514a (Pri-miR-514a) is transcribed and processed into pre-miR-514a. Pre-miR-514a is further processed into two mature forms: miR-514a-5p and miR-514a-3p. Mature sequences of miR-514a-5p and miR-514a-3p are indicated in green and blue, respectively, and their seed regions are highlighted. **(B–D)** Expression levels of the mature forms of miR-514a were measured by qRT-PCR at each stage of neuronal differentiation from iPSCs (201B7, 585A1 and 648A1) to neurons. *n* = 3 independent experiments. ***p < 0.001, **p < 0.01, *p < 0.05. Abbreviations: pre-miR-514a-1, precursor miR-514a-1; pre-miR-514a-2, precursor miR-514a-2; pre-miR-514a-3, precursor miR-514a-3; iPSC, induced pluripotent stem cell.

miR-514a is a member of a cluster of miRNAs on chrXq27.3 and is encoded at three genomic loci, MIR514A1, MIR514A2, and MIR514A3, all of which generate two types of mature forms, miR-514a-5p and miR-514a-3p (Yoshida et al., 2021) (Figure 1A). Although miR-514a has been well studied in tumor cells (Liu et al., 2014; Xiao et al., 2018), function of miR-514a in the nervous system has not been elucidated. Additionally, miR-514a is expressed in highly social species, such as humans and chimpanzees, but not in rodents. To explore the possible roles of miR-514a in nervous system development, we first investigated the expression levels of miR-514a at each stage of neuronal differentiation in healthy human subject-derived iPSC lines (201B7, 585A1, and 648A1). The expression level of mature miR-514a increased when iPSCs differentiated into neurospheres and showed a tendency to increase further when differentiated into neurons (Figures 1B–D). These results suggest that miR-514a plays a role in neuronal development.

### 3.2 miR-514a promotes neuronal differentiation and dendritic development in iPSC-derived neurons

**FIGURE 2:**
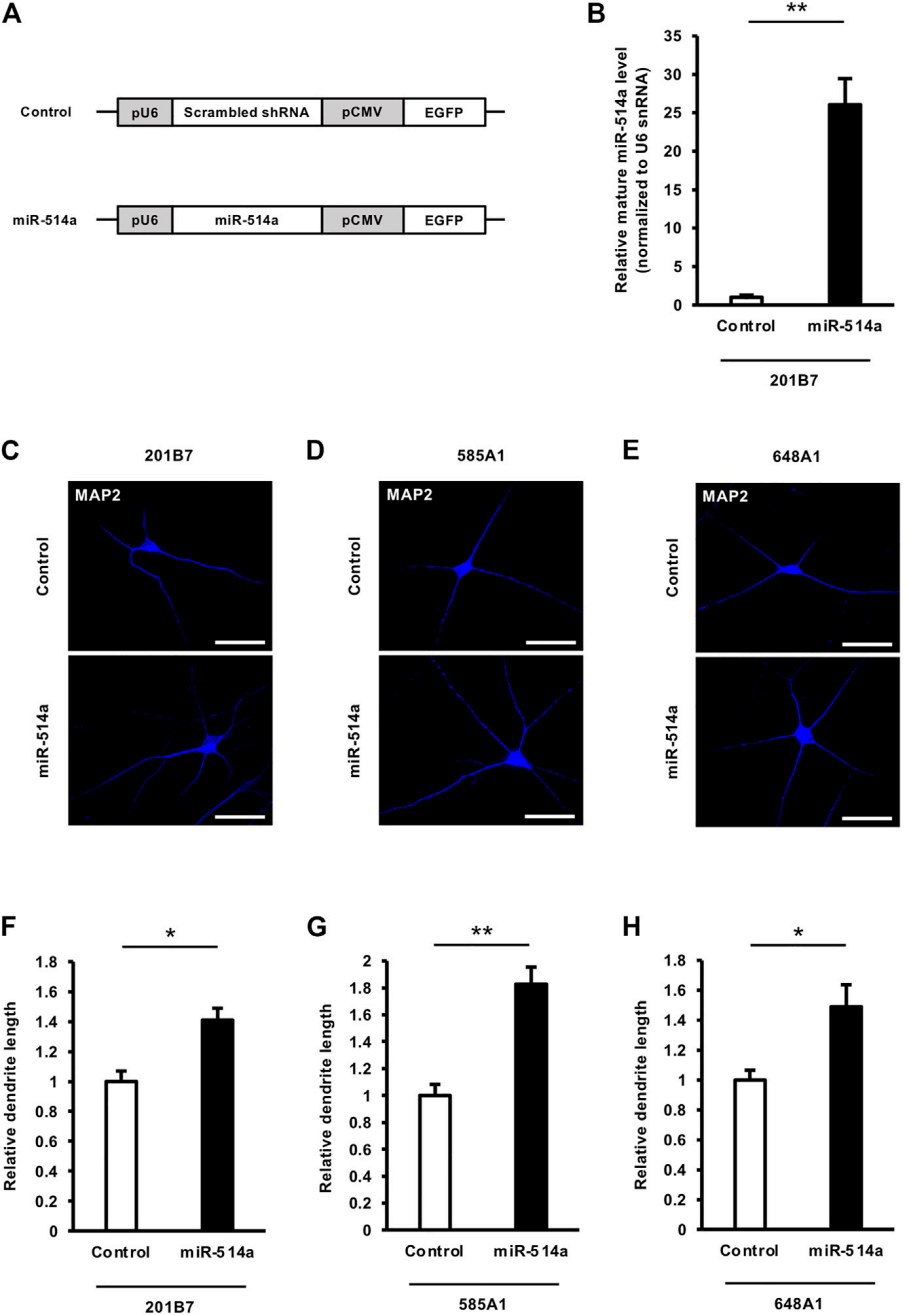
miR-514a promotes dendritic development in human iPSC-derived neurons. **(A)** Diagrams of lentiviral vector constructs. miR-514a is expressed as a short hairpin under the U6 polymerase - promotor, and EGFP is expressed under the CMV promotor. **(B)** Expression levels of mature miR-514a were confirmed using qRT-PCR. 201B7 iPSC-derived neurons were infected with lentiviruses expressing scrambled shRNA and miR-514a, respectively, and cultured for 7 days and subsequently lysed for qRT-PCR. *n* = 3 independent experiments. **(C–E)** Representative images of iPSC-derived neurons stained with anti-MAP2 (blue) antibody at 7 DIV. Scale bars, 50 µm. **(F–H)** Quantitative analysis of total dendrite length of neurons derived from three independent iPSC line, respectively. *n* = 3 independent experiments, at least 100 neurons were analyzed in each experiment. **p < 0.01, *p < 0.05. Abbreviations: CMV, cytomegalovirus; EGFP, enhanced green fluorescent protein; MAP2, microtubule-associated protein 2.

The proper regulation of neurogenesis is essential for the brain development and the brain function. Recent studies have been shown miRNAs are implicated in neurogenesis (Shi et al., 2010; Lang and Shi, 2012). To investigate whether miR-514a regulates neuronal differentiation, we expressed miR-514a in iPSCs (201B7, 585A1, and 648A1) at the terminal induction step into neuronal differentiation with a lentivirus expressing miR-514a or scrambled shRNA as control (Figure 2A) and evaluated the rate of neuronal differentiation. Overexpression of miR-514a was confirmed using qRT-PCR in cultured neurons (Figure 2B). Seven days after lentiviral infection, differentiated neurons were examined by immunocytochemistry using anti-Tuj-1 and anti-MAP2 antibodies. We found that the percentage of Tuj-1 positive neurons and MAP2 positive neurons in miR-514a expressing iPSCs were significantly higher than control (Supplementary Figures S1A–I). These data suggest that miR-514a expression enhance neuronal differentiation of cultured human iPSCs.

Since dendrites play a critical role in synaptic input, the regulation of their growth is important for the function of neuronal networks (Spruston, 2008). Thus, dendritic abnormalities in neurons are highly correlated with neurological disorders, such as mental retardation and aging diseases (Kaufmann and Moser, 2000; Penzes et al., 2011). To investigate the function of miR-514a in neurons, we expressed miR-514a in neurons differentiated from the human iPSC 201B7 line by infecting them with a lentivirus expressing miR-514a. Overexpression of miR-514a in neurons significantly increased the total dendritic length compared to the control (Figures 2C, F). Similar results were obtained for the regulatory function of miR-514a in dendrites from the other two human iPSC lines (585A1 and 648A1) (Figures 2D, E, G, H). Our results suggested that miR-514a plays an important role in the regulation of dendritic development in neurons derived from multiple human iPSC lines.

### 3.3 miR-514a increases neuronal soma size

**FIGURE 3:**
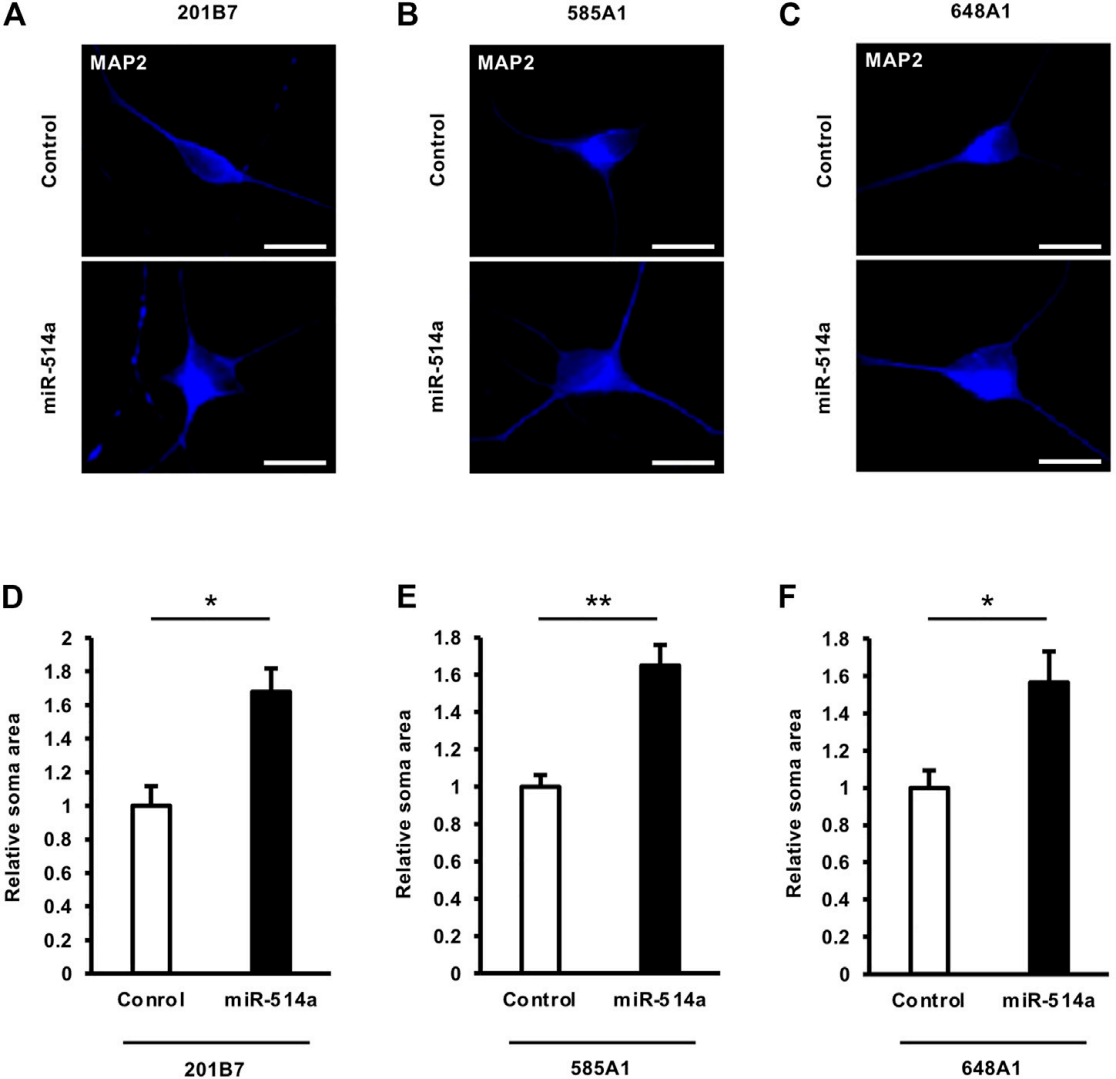
miR-514a increases neuronal soma size. Neurons were infected with lentiviruses expressing miR-514a and cultured for 7 days. **(A–C)** Representative images of iPSC-derived neurons stained with anti-MAP2 (blue) antibody at 7 DIV. Scale bars, 25 µm. **(D–F)** Quantitative analysis of neuronal soma size of neurons derived from three independent iPSC line, respectively. *n* = 3 independent experiments, at least 100 neurons were analyzed in each experiment. **p < 0.01, *p < 0.05. Abbreviations: MAP2, microtubule-associated protein 2.

The control of neuronal soma size, which reflects neuronal cell growth, is important for proper brain circuit function, and its dysregulation is linked to the pathogenesis of several neurodevelopmental disorders, including autism spectrum disorder (ASD), intellectual disability (ID) and attention deficit hyperactivity disorder (ADHD) (Parenti et al., 2020). To investigate whether miR-514a could influence neuronal soma size, we expressed miR-514a in cultured neurons derived from the human iPSC 201B7 line by infecting them with a lentivirus expressing miR-514a. Image analyses indicated that overexpression of miR-514a resulted in significantly increased neuronal soma size in iPSC-derived human neurons compared with the control (Figures 3A, D). This result was confirmed in other iPSC lines, 585A1 and 648A1 (Figures 3B, C, E, F), suggesting that miR-514a positively regulates neuronal cell growth.

### 3.4 miR-514a upregulates mTOR signaling pathway

**FIGURE 4:**
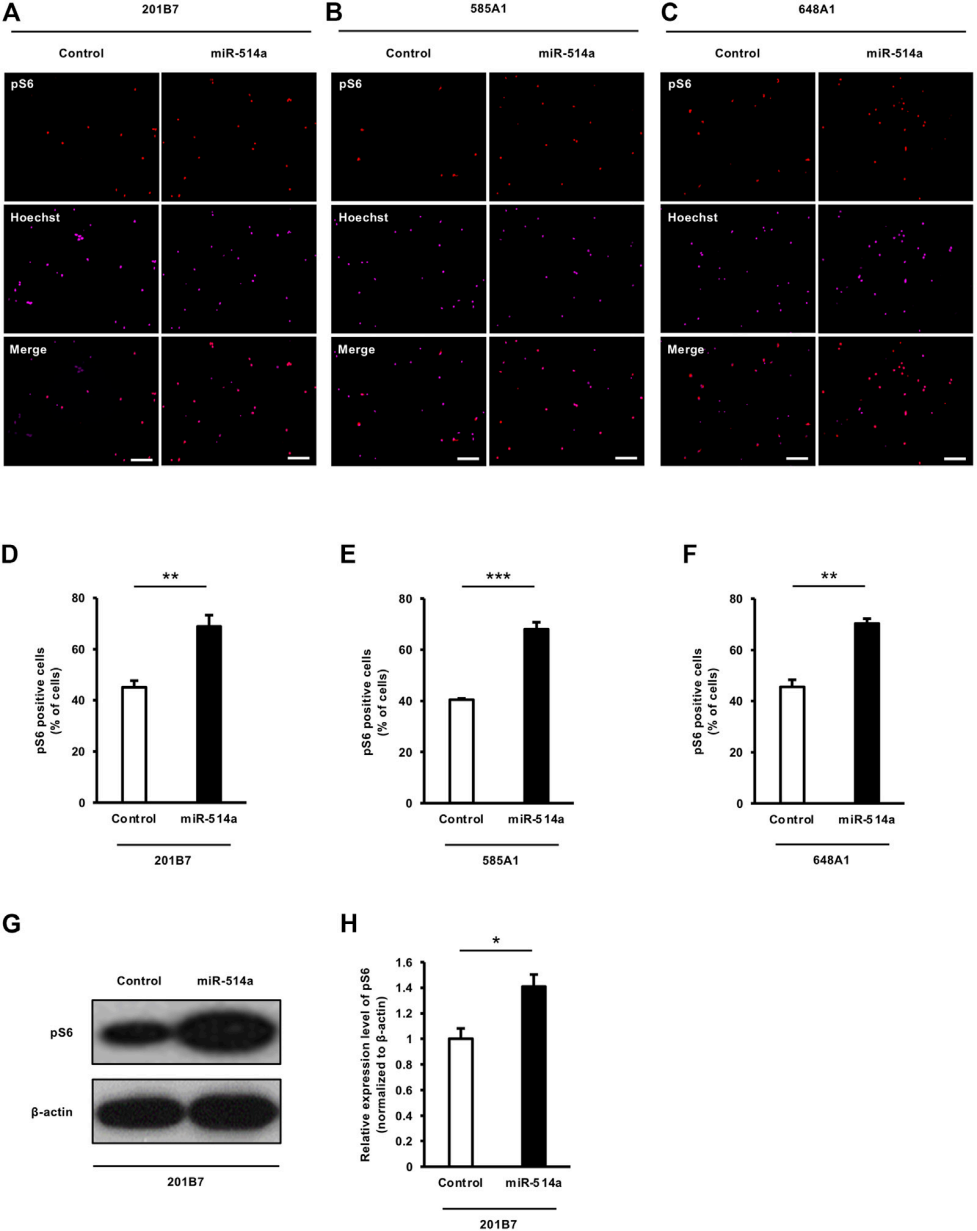
miR-514a upregulates mTOR signaling pathway in human iPSC-derived neurons. Neurons were infected with lentiviruses expressing miR-514a and cultured for 7 days. **(A–C)** Representative images of iPSC-derived neurons stained with anti-pS6 (red) antibody and Hoechst 33,258 (magenta) at 7 DIV. Scale bars, 100 µm. **(D–F)** pS6 positive neurons derived from three independent iPSC line, respectively, were quantified. mTOR activity was markedly increased in neurons expressed miR-514a. *n* = 3 independent experiments, at least 200 neurons were analyzed in each experiment. **(G)** Western blotting analysis showing the level of pS6 in cultured neurons at 7 DIV. **(H)** Quantification of pS6 level. *n* = 3 independent experiments. ***p < 0.001, **p < 0.01, *p < 0.05. Abbreviations: pS6, phospho-S6 ribosomal protein.

Several studies have shown that the mTOR pathway is involved in the regulation of cell growth in mammalian cells, including neurons, and positively controls the soma size of the cells (Lipton and Sahin, 2014). Since miR-514a increased neuronal soma size, we speculated that miR-514a might regulate mTOR signaling activity in neurons. To examine the effect of miR-514a on mTOR signaling, we expressed miR-514a in 201B7 iPSC-derived neurons and immunostained them with anti-pS6 antibody as a marker of mTOR activity. Quantitative analysis revealed that the overexpression of miR-514a increased the percentage of pS6 positive neurons (Figures 4A, D). Western blotting analysis revealed a marked increase of pS6 level following the expression of miR-514a (Figures 4G, H). Similarly, we confirmed that miR-514a upregulates mTOR signaling in neurons derived from two other iPSC lines (585A1 and 648A1) (Figures 4B, C, E, F).

### 3.5 Inhibition of miR-514a suppresses neuronal development in iPSC-derived neurons

**FIGURE 5:**
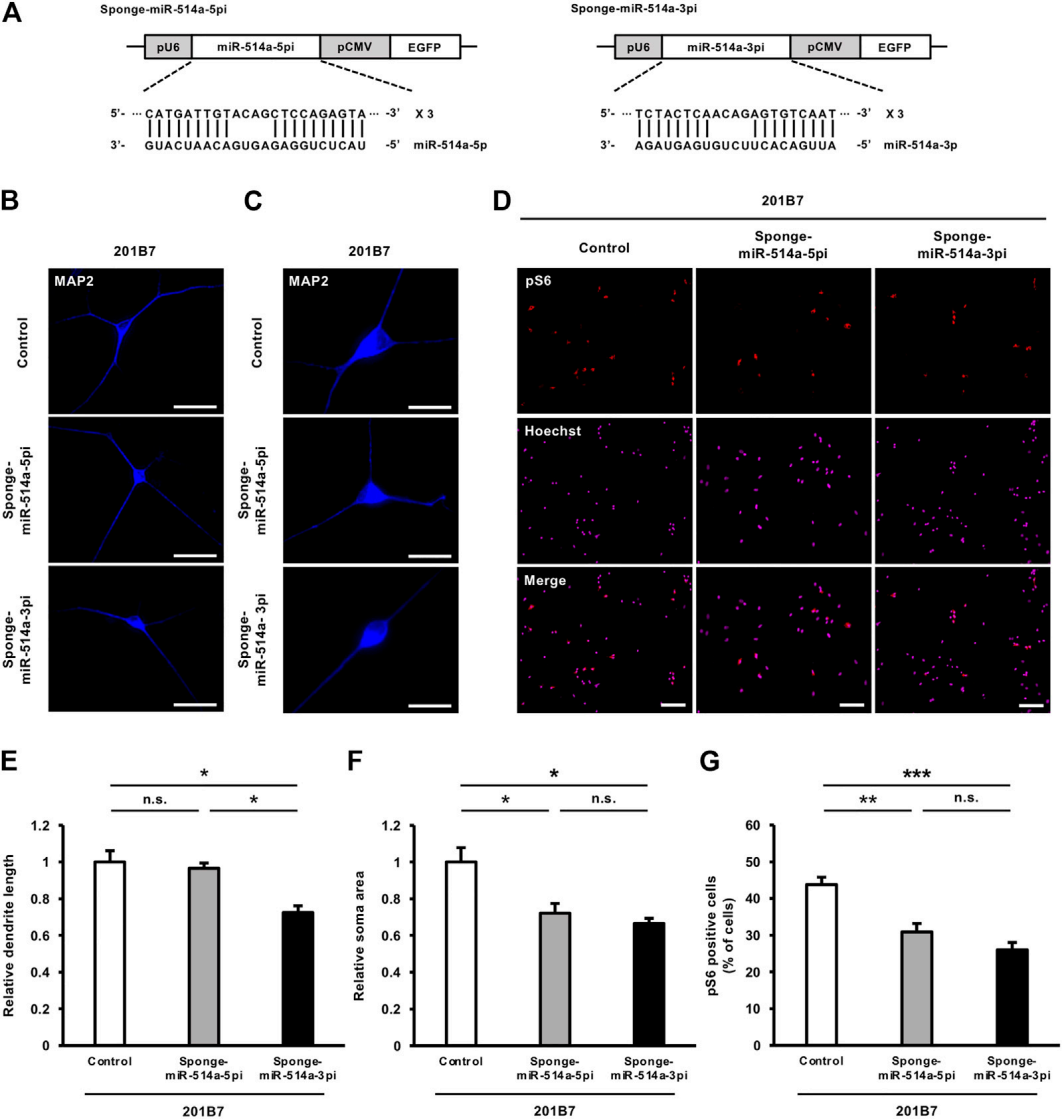
Blocking miR-514a using its sponges causes inhibition of neuronal development in iPSC-derived neurons. Neurons were infected with lentiviruses expressing miR-514a-5p sponge, miR-514a-3p sponge or scrambled shRNA as control and cultured for 7 days. **(A)** Diagrams of lentiviral vector constructs expressing sponges against miR-514a-5p and miR-514a-3p. The sponges were expressed under the U6 RNA polymerase Ⅲ promotor, and EGFP is expressed under the CMV promotor. **(B)** Representative images of iPSC-derived neurons (201B7) stained with anti-MAP2 (blue) antibody at 7 DIV. Scale bars, 50 µm. **(C)** Representative images of iPSC-derived neurons (201B7) stained with anti-MAP2 (blue) antibody at 7 DIV. Scale bars, 25 µm. **(D)** Representative images of iPSC-derived neurons (201B7) stained with anti-pS6 (red) antibody and Hoechst 33,258 (magenta) at 7 DIV. Scale bars, 100 µm. **(E)** Quantitative analysis of total dendrite length. *n* = 3 independent experiments, at least 100 neurons were analyzed in each experiment. **(F)** Quantitative analysis of neuronal soma size. *n* = 3 independent experiments, at least 100 neurons were analyzed in each experiment. **(G)** Quantitative analysis of pS6 positive cells. *n* = 3 independent experiments, at least 200 cells were analyzed in each experiment. *n* = 3 independent experiments. ***p < 0.001, **p < 0.01, *p < 0.05. Abbreviations: CMV, cytomegalovirus; EGFP, enhanced green fluorescent protein; MAP2, microtubule-associated protein 2; pS6, phospho-S6 ribosomal protein; n. s., not significant.

We next investigated whether the loss of miR-514a function leads to the opposite effect induced by miR-514a overexpression in iPSC-derived neurons. We applied a loss-of-function approach by expressing miR-514a sponges. A miRNA sponge is an RNA transcript which contains multiple target sites complementary to a mature miRNA. It can specifically bind to endogenous miRNAs and inhibit their silencing activity (Ebert et al., 2007). Pre-miR-514a generates two mature miRNAs, miR-514a-5p and miR-514a-3p. Therefore, we designed a miRNA sponge against each miR-514a-5p and miR-514a-3p, which consists of four narrowly spaced, bulged binding sites for mature forms (Figure 5A). We then expressed miR-514a sponge in cultured neurons by infecting them with a lentivirus expressing miR-514a sponge, respectively. Inhibition of endogenous miR-514a-3p significantly reduced dendritic development, whereas the miR-514a-5p inhibition did not affect dendritic development (Figures 5B, E). Moreover, blocking of either miR-514a-5p or miR-514a-3p functions decreased neuronal soma size (Figures 5C, F) and downregulated mTOR signaling pathway in each case (Figures 5D,G H and I). To gain insight into mechanism how miR-514a exert functions, we further examined the downstream target genes of miR-514a-5p and 3p by *in silico* miRNA target gene prediction database (TargetScan: https://www.targetscan.org/vert_80/). We found that miR-514a-3p potentially targets several endogenous mTOR signal inhibitors such as tuberous sclerosis 1 (TSC1), phosphatase and tensin homolog (PTEN), DEP domain containing MTOR-interacting protein (DEPTOR) (Gao et al., 2011), whereas miR-514a-5p potentially targets only TSC1 but not PTEN and DEPTOR (Supplementary Tables S1, S2). These predictions support the stronger effect of miR-514a-3p. We also explored other potential target genes of miR-514a by using another database, miRTarBase (The experimentally validated microRNA-target interactions database: https://mirtarbase.cuhk.edu.cn/∼miRTarBase/miRTarBase_2022/php/index.php) (Supplementary Table S3). However, we could not find the potential miR-514a targets genes belonging to the mTOR signaling pathway. In addition to these explorations of miR-514a targets, a previous study has reported that miR-514a targets NF1, an endogenous mTOR signal inhibitor (Stark et al., 2015)*.* Taken together, these results indicate that inhibition of miR-514a suppresses neuronal development in human iPSC-derived neurons.

### 3.6 A variation in miR-514a sequences among primate genomes affects the expression level of mature miR-514a

**FIGURE 6:**
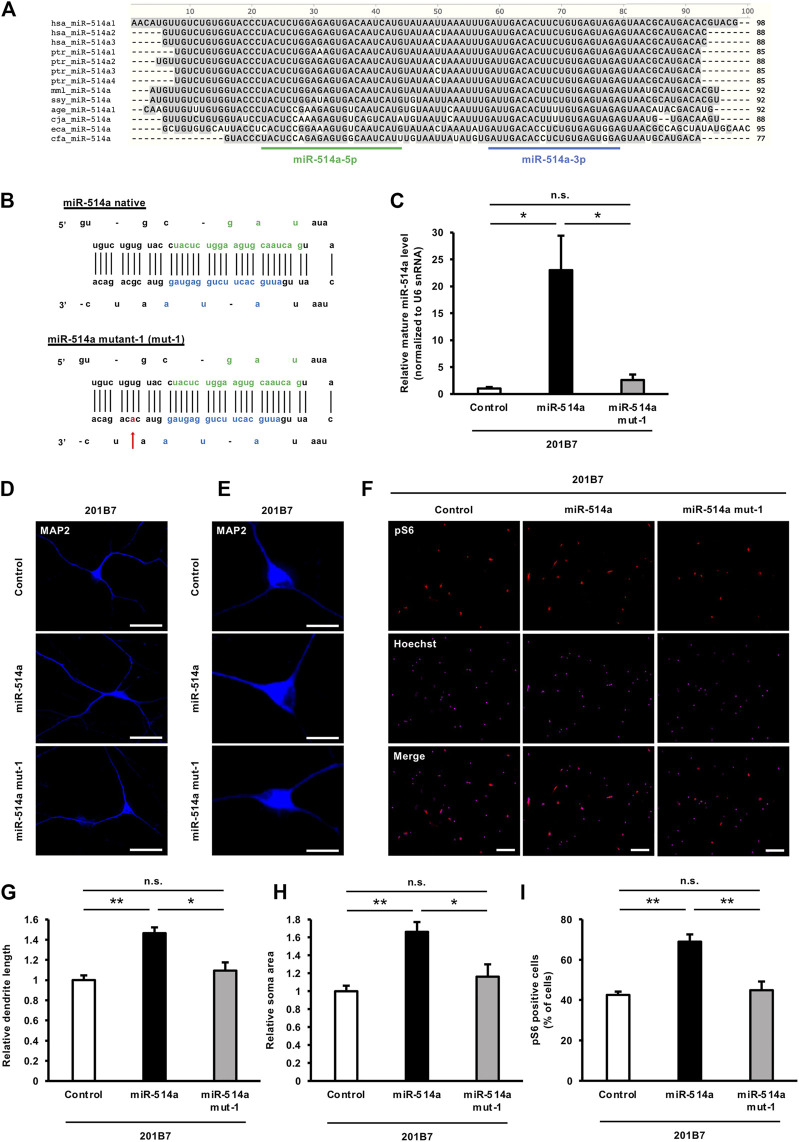
Variation in miR-514a sequences among primate genomes affects the expression level of mature miR-514a. **(A)** Comparison of sequence of precursor-miR-514a across species. **(B)** Schematic representation of the mutation in pre-miR-514a-3. (the nucleotide position in the mutation is indicated with arrow). **(C)** Expression levels of mature miR-514a were measured using qRT-PCR in 201B7 iPSC-derived neurons infected with lentiviruses expressing native miR-514a, miR-514a mutant or scrambled shRNA as control. *n* = 3 independent experiments. **(D)** Representative images of iPSC-derived neurons stained with anti-MAP2 (blue) antibody at 7 DIV. Scale bars, 50 µm. **(E)** Representative images of iPSC-derived neurons stained with anti-MAP2 (blue) antibody at 7 DIV. Scale bars, 25 µm. **(F)** Representative images of iPSC-derived neurons stained with anti-pS6 (red) antibody and Hoechst 33,258 (magenta) at 7 DIV. Scale bars, 100 µm. **(G)** Quantitative analysis of total dendrite length. *n* = 3 independent experiments, at least 100 neurons were analyzed in each experiment. **(H)** Quantitative analysis of neuronal soma size. *n* = 3 independent experiments, at least 100 neurons were analyzed in each experiment. **(I)** Quantitative analysis of pS6 positive cells. *n* = 3 independent experiments, at least 200 cells were analyzed in each experiment. **p < 0.01, *p < 0.05. Abbreviations: MAP2, microtubule-associated protein 2; pS6, phospho-S6 ribosomal protein; n. s., not significant.

Given that mTOR signaling has been implicated in a variety of biological processes and disease pathogenesis in the nervous system, we reasoned that the different levels of mTOR signaling activity across its physiological range during evolution may affect species-specific brain development and function. Since we have shown in this study that miR-514a regulates mTOR signaling, we decided to further investigate the relationship between miR-514a expression and the evolution of nervous system development. Several studies have reported that variations in precursor and mature miRNA sequences influence the final mature miRNA biogenesis (Sun et al., 2009; Reichenstein et al., 2019). Therefore, we first compared the precursor- and mature-miR-514a sequence across species (Figure 6A). We found that the precursor- and mature-miR-514a sequences were highly conserved in primate genomes, whereas many variations were observed in lower species such as horses and dogs. In particular, we focused on variations found among primate genomes to assess whether they affect the acquisition of high intelligence and sociality during evolution. Next, to examine the effect of interspecies variations on miR-514a expression, we generated expression vectors expressing the pre-miR-514a mutant harboring mutations found among primates (Supplementary Figure S2A). The expression levels of mature-miR-514a were assessed by qRT-PCR (Supplementary Figure S2B). We found that the mutant expression vector harboring mutations in the stem region of pre-miR-514a significantly reduced the expression level of mature-miR-514a. Notably, designed mutant-1 (Figure 6B) resulted in markedly decreased level of mature form by 10-fold compared to native miR-514a (Figure 6C). Furthermore, expression of the miR-514a mutant-1 failed to enhance dendrite outgrowth, neuronal soma size, and mTOR signaling activity in iPSC-derived neurons (Figures 6D–I). These results suggest that miR-514a variation during evolution can affect neurodevelopment and function.

## 4 Discussion

In recent decades, miRNAs have been shown to regulate various biological processes (Ambros, 2004; Bartel, 2004). Numerous studies have provided evidence for the crucial role of miRNAs in brain development and function (Saba and Schratt, 2010; Im and Kenny, 2012; Sun and Shi, 2015). Dysregulation of miRNA expression and function has been reported to cause developmental abnormality and is implicated in the pathogenesis of a variety of neurological disorders (Bartel, 2018; Juzwik et al., 2019). In the present study, we demonstrated that overexpression of miR-514a increased total dendritic length and soma size, and upregulated the mTOR signaling pathway in human iPSC-derived neurons. These data indicate that miR-514a positively regulates neuronal development. To the best of our knowledge, this study is the first to report the functional roles of miR-514a in neuronal development.

Non-conserved miRNAs play important roles in various species-specific biological functions and disease pathologies. Several species-specific miRNAs, such as miR-941 and miR-95, have been reported to play key roles in certain biological processes and pathogenesis of disease (Hu et al., 2012; Frankel et al., 2014). Given that the number of duplications miR-514a has undergone throughout primate evolution, it is possible that miR-514a plays a role in primate brain evolution and contributes to higher brain functions, such as social behavior, cognition, and decision-making, which are associated with the pathophysiology of developmental disorders. To test this possibility, it is not sufficient to analyze the expression level of miRNA alone, and it is necessary to elucidate its functional role in neurons. In this study, we showed that miR-514a expression promotes dendritic formation and soma size. These results suggest that miR-514a positively regulates neuronal development and that increased levels of miR-514a may be involved in the evolution of brain function. Future *in vivo* studies using non-human primate models will provide more compelling findings.

The dendrites that receive information inputs from other neurons and the dendrite morphology have important functional implications in determining what signals a neuron receives and how these signals are integrated, and the regulation of dendrite growth is critical for the establishment of functional neuronal networks (Craig and Banker, 1994; Spruston, 2008). Defects in dendrite development or maintenance could contribute to neurological and neurodevelopmental disorders such as schizophrenia, fragile X syndrome, Angelman syndrome, and Rett syndrome (Garey et al., 1998; Kaufmann and Moser, 2000; Armstrong, 2005; Dindot et al., 2008). Several specific miRNAs have been reported to act as regulators of dendritic development and are involved in dendritic phenotypes in diseases. For example, miR-214 promotes dendritic development by targeting the schizophrenia-associated gene QKI (Irie et al., 2016), and miR-138 restricts the size of dendritic spines by regulating APT1 (Siegel et al., 2009). Our finding that miR-514a promotes dendritic development provides important data for an expanded understanding of miRNAs as key players in functional neuronal networks.

mTOR, a highly conserved serine/threonine kinase expressed in most mammalian cell types, plays a central role in cell growth, proliferation, survival, and metabolism (Laplante and Sabatini, 2012). Growing evidence indicates that the mTOR signaling pathway is an essential regulator of neuronal development, survival, differentiation, and synaptic plasticity (Jaworski and Sheng, 2006; Meng et al., 2018), and the mTOR signaling pathway is also associated with the pathogenesis of a broad range of neurological diseases, including neurodevelopmental disorders and neurodegenerative diseases (Costa-Mattioli and Monteggia, 2013; Perluigi et al., 2015). Several recent studies have indicated that miRNAs are involved in regulating mTOR signaling by targeting multiple components of this signaling pathway. miR-212-3p improves the functional recovery of spinal cord injury and inhibits lipopolysaccharide-induced neurocyte apoptosis by targeting PTEN to activate the AKT/mTOR pathway (Guan et al., 2021). miR-193b-3p is significantly downregulated in patients with amyotrophic lateral sclerosis (ALS) (Chen et al., 2016) and downregulation of miR-193b-3p promotes autophagy and cell survival by targeting TSC1/mTOR signaling (Li et al., 2017). In a microarray study of patients with cortical dysplasia, 10 differentially expressed miRNAs were upregulated, and the mTOR signaling pathway was the most significantly associated (Lee et al., 2014). Our previous study showed that miR-199a positively controls mTOR signaling activity by suppressing mTOR signaling inhibitors such as Pde4d, Sirt1, and Hif1α downstream of methyl-CpG-binding protein 2 (MeCP2), which is a causative gene for the severe neurodevelopmental disorder Rett syndrome (Tsujimura et al., 2015). Given the contribution of the mTOR pathway to nervous system development and disease pathogenesis, miRNA-mediated regulation of the mTOR pathway may play a critical role in brain development and various neurological disease states. Here, we have shown that miR-514a upregulates the mTOR signaling pathway in iPSC-derived neurons and an miR-514a mutant harboring mutations found among primates diminishes the mTOR signal-enhancing effect observed in native miR-514a. These findings further support the importance of miRNAs in nervous system development and brain evolution, which is critical for the acquisition of higher mental functions, and suggest that miR-514a plays a key role in brain development and disorders.

As a single miRNA can target numerous different genes and simultaneously regulate different pathways, miRNAs are thought to have a profound impact on the development of organisms and diseases. Although previous studies have reported that miR-514a targets NF1 and EGFR genes (Stark et al., 2015; Ke et al., 2017), many potential target genes of miR-514a have not yet been identified. Future research examining the downstream target genes of miR-514a would provide further insight into the regulatory mechanisms of miR-514a in neuronal development.

In summary, this study revealed an important role of miR-514a in neuronal development and its possible involvement in the evolution of brain development. We expect that more detailed elucidation of downstream pathway of miR-514a will improve the understanding of the molecular mechanisms underlying the evolution of brain function, CNS development, and disease pathologies associated with neurodevelopmental abnormalities.

## Data Availability

The original contributions presented in the study are included in the article/Supplementary Material, further inquiries can be directed to the corresponding authors.

## Supplementary material

SUPPLEMENTARY FIGURE S1 miR-514a promotes neuronal differentiation of human iPSCs. iPSCs (201B7, 585A1, and 648A1) were cultured in differentiation medium and lentivirus expressing miR-514a was transduced at the induction step into neuronal differentiation. Differentiated neurons were examined by immunocytochemistry using anti-Tuj-1 and anti-MAP2 antibodies. **(A–C)** Representative images of iPSC-derived neurons stained with anti-Tuj-1 (green) antibody, anti-MAP2 (blue) antibody and Hoechst 33258 (magenta) at 7 DIV. Scale bars, 150 µm. **(D–F)** Quantitative analysis of Tuj-1 positive neurons. *n* = 3 independent experiments, at least 200 neurons were analyzed in each experiment. **(G–I)** Quantitative analysis of MAP2 positive neurons. *n* = 3 independent experiments, at least 200 neurons were analyzed in each experiment. ***p < 0.001, **p < 0.01, *p < 0.05. Abbreviations: Tuj-1, β-Ⅲ tubulin; MAP2, microtubule-associated protein 2; pS6, phospho-S6 ribosomal protein.

SUPPLEMENTARY FIGURE S2 miR-514a mutations found in cross-species sequence comparison attenuate mature-miR-514a expression. **(A)** Mutation profile of precursor-miR-514a (the nucleotide positions in the mutations are indicated with arrows). Mutant-1 (G > A) and -5 [C > U(T)] were located in the stem region of pre-miRNA sequence, mutant-2 [U(T) > C] and -4 [U(T) > C] were harbored within the nucleotides of mature miRNA and mutant-3 (A > G) was located in the loop region of pre-miRNA. These variants are single-nucleotide substitutions that are conserved in humans and chimpanzees but not in other primates, dogs, or horses. **(B)** The expression levels of mature-miR-514a in mutants detected by qRT-PCR. ****p < 0.0001. Abbreviations: n.s., not significant.
