# Supplementary figures and images for: miR-514a promotes neuronal development in human iPSC-derived neurons
Source: Front Cell Dev Biol. 2023 Feb 7;11:1096463. doi: 10.3389/fcell.2023.1096463 (PMC9941156; doi:10.3389/fcell.2023.1096463)

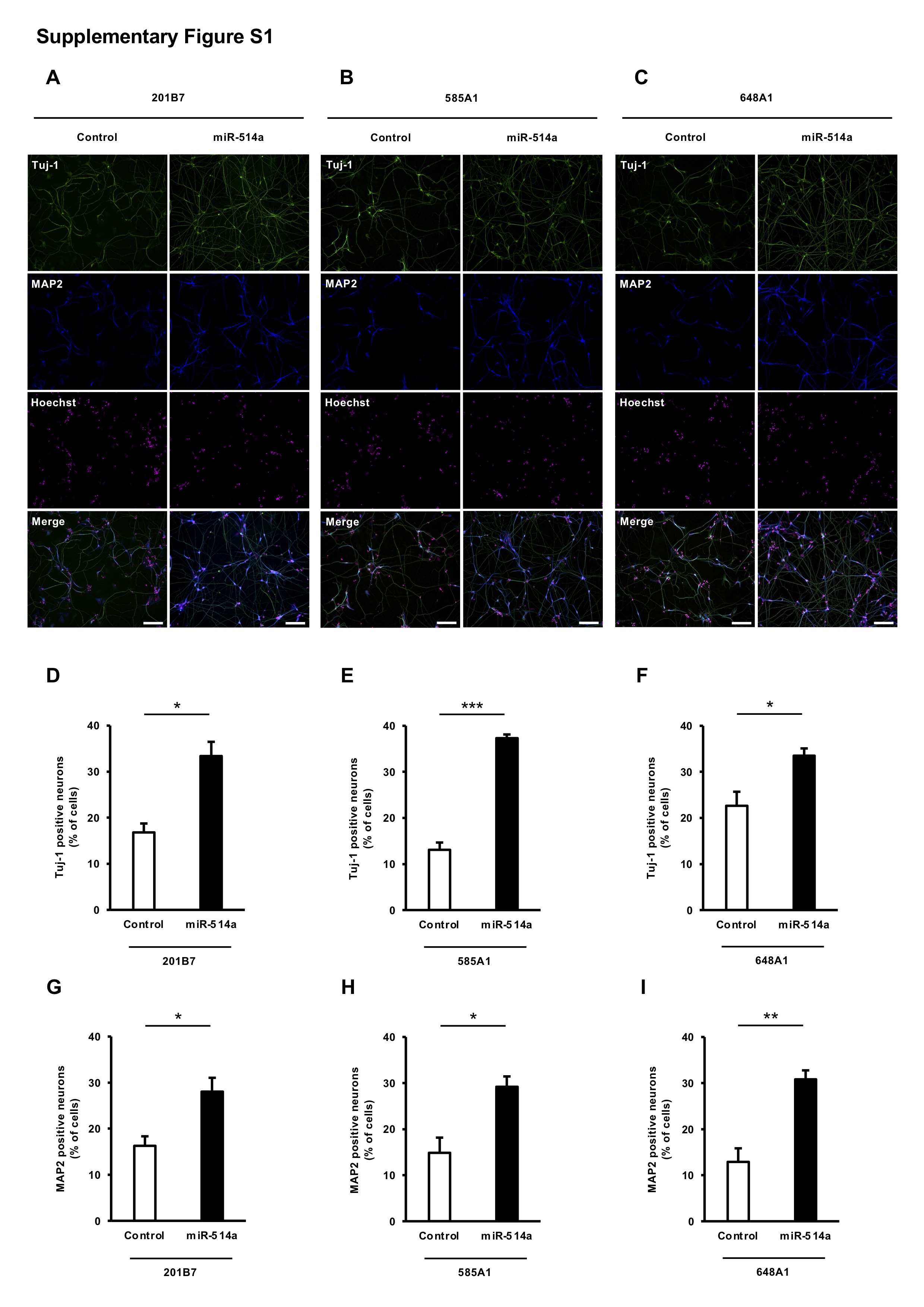

Supplement: Supplementary file 3 [file Image1.JPEG]

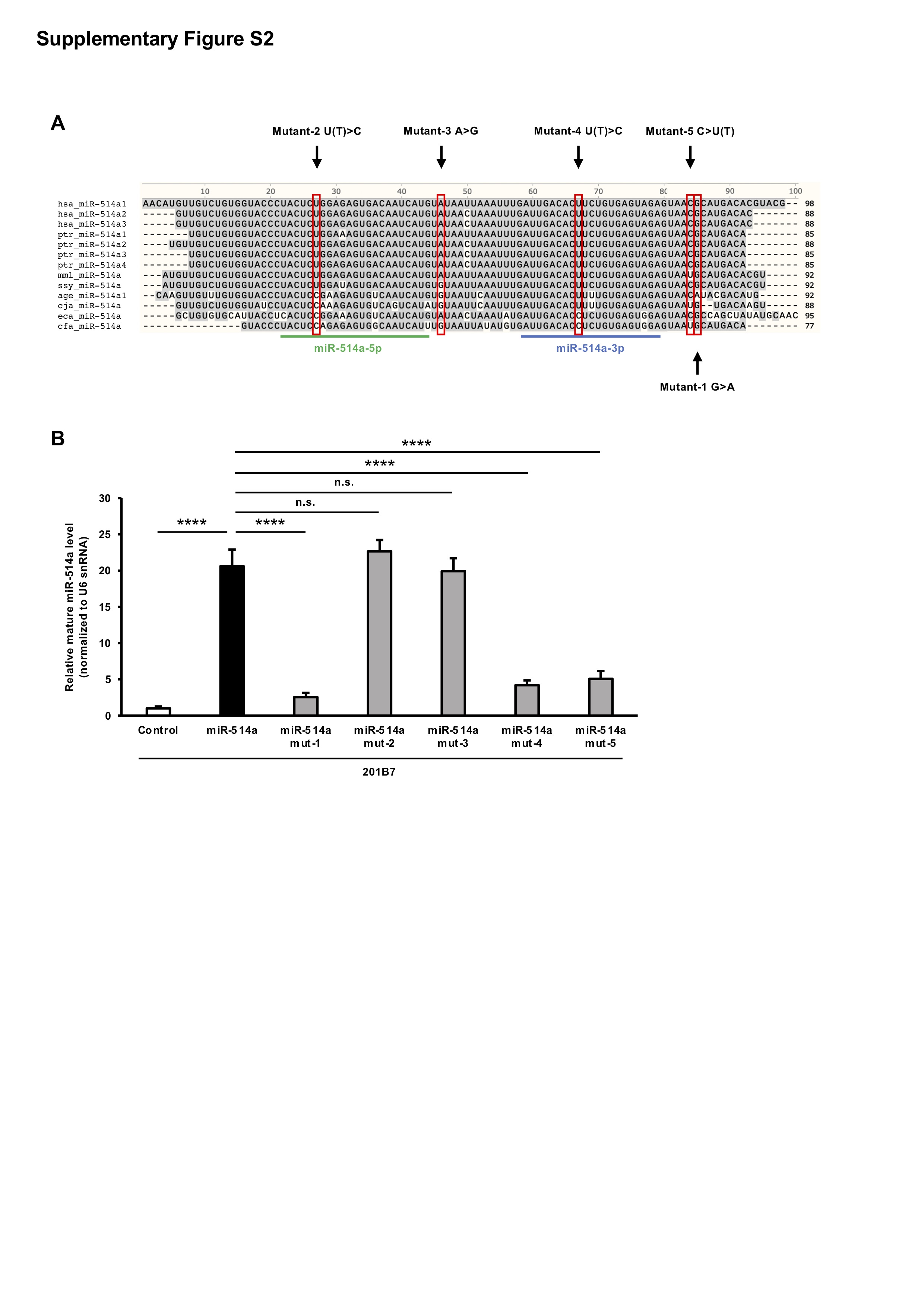

Supplement: Supplementary file 4 [file Image2.JPEG]
